# Theory-guided multifunctional Zn-Salen molecular catalyst for sustainable polyester plastic recycling

**DOI:** 10.1039/d5sc04667j

**Published:** 2025-08-25

**Authors:** Mei Li, Yawen Shi, Lei Tang, Na Ji, Shengbo Zhang

**Affiliations:** a School of Environmental Science and Engineering, Tianjin Key Laboratory of Biomass/Wastes Utilization, Tianjin University Tianjin 300350 China jina@tju.edu.cn shengbozhang@tju.edu.cn; b School of Materials Science and Engineering, Smart Sensing Interdisciplinary Science Center, Nankai University Tianjin 300350 China; c Institute of Sustainability for Chemicals, Energy and Environment (ISCE2), Agency for Science, Technology and Research (A*STAR) 1 Pesek Road Singapore 627833 Republic of Singapore Tang_Lei@isce2.a-star.edu.sg

## Abstract

The escalating global challenge of plastic waste calls for innovative recycling solutions that overcome the high energy requirements of traditional chemical recycling and the inefficiency of enzymatic methods. Here, inspired by the structure of Salen-based molecular catalysts and the hydrolase-mediated degradation mechanism of poly(ethylene terephthalate) (PET), we report a multifunctional Zn-Salen molecular catalyst identified through theoretical screening and experimental validation. This catalyst achieves high PET conversion efficiency under mild conditions with low energy consumption. Mechanistic investigations reveal that its Zn metal site and quaternary ammonium component synergistically promote adsorption, activation, and nucleophilic attack towards the O–C=O group in PET *via* a proximity effect, mimicking key features of PET hydrolases. Notably, the synthetic catalyst demonstrates high resistance to external interference, achieving an industrially viable productivity of 75 g_TPA_ L^−1^ h^−1^ at *C*_OH−_ = 0.1 M and *T* = 90 °C. This catalyst is also effective for diverse substrates, including real-world PET waste, mixed plastics containing PET, and biodegradable PLA. Techno-economic and environmental analyses indicate that this recycling system can significantly reduce carbon emissions and has potential commercial value.

## Abstract 2

A multifunctional Zn-Salen catalyst achieves high PET conversion under mild conditions. Zn site and quaternary ammonium component promote activation and nucleophilic attack towards the O–C=O *via* a proximity effect.

## Graphical abstract

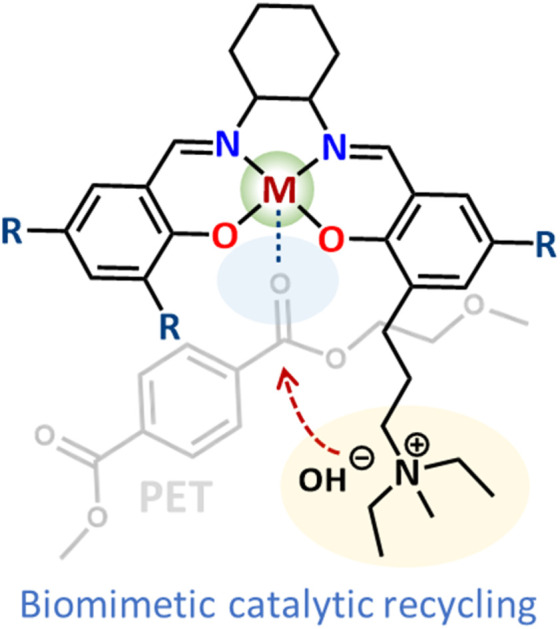

## Introduction

**Fig. 1:**
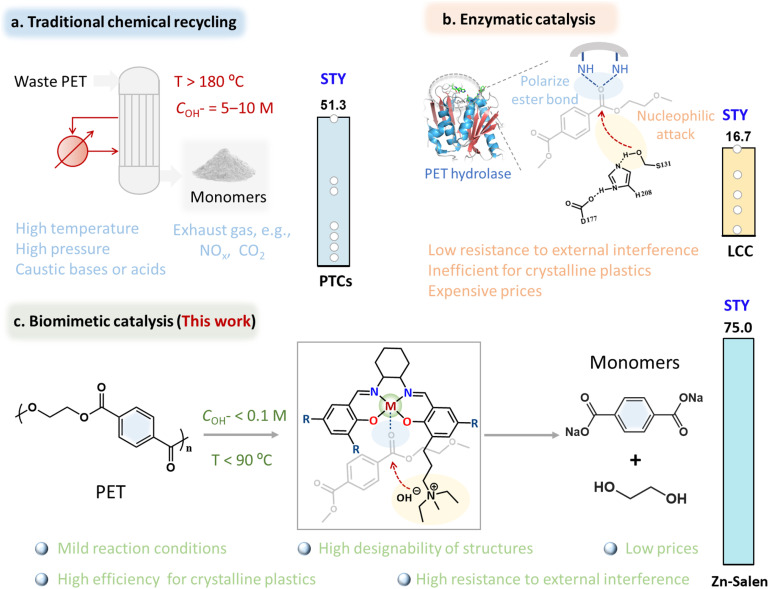
Plastic recycling methods and Salen-based molecular catalyst design. (a) Traditional chemical recycling process, STY: space-time yield, PTCs: phase transfer catalysts. (b) PET hydrolase catalysis (LCC: leaf-branch compost cutinase) and (c) biomimetic catalysis (this work). Schematic representations of the active sites and reaction mechanisms proposed for PET hydrolase (b) and the hypothesized PET-degrading multifunctional Salen-based molecular catalyst (c), where M represents metal sites, R represents substituent functional groups, gray arcs represent active-site backbones. Note that the metal center and quaternary ammonium salt component play critical roles in accelerating ester hydrolysis *via* the organization of substrates in close proximity.

The exponential growth of plastic production since the 1950s has led to widespread contamination of ecosystems, causing significant environmental harm.^1–6^ Among various plastics, poly(ethylene terephthalate) (PET) stands out as the most widely produced polyester, accounting for approximately 12% of global solid waste.^7–9^ While mechanical recycling methods often degrade PET's material properties,^10,11^ chemical recycling offers the advantage of restoring its original functionality. However, chemical recycling typically requires extreme conditions, including high temperatures, pressures, and corrosive reagents, resulting in high energy consumption and hazardous waste generation (Fig. 1a).^10,12^ These limitations underscore the urgent need for innovative recycling methods that combine efficiency with environmental sustainability.

Enzymatic PET depolymerization has emerged as a promising alternative, offering the potential to recycle plastic waste under mild, environmentally friendly conditions.^13–18^ Enzymes such as PETases and cutinases can break down PET into its monomers-terephthalic acid (TPA) and ethylene glycol (EG)-at moderate temperatures and neutral pH. However, despite substantial advances in enzyme engineering, these methods are still hindered by low conversion efficiency and sensitivity to external factors such as temperature, pH, and the presence of additives.^10,19,20^ These limitations significantly restrict their scalability and practical application. Consequently, there is an urgent need to develop strategies that address the energy-intensive nature of chemical recycling while overcoming the efficiency and robustness challenges of enzymatic depolymerization.

A key mechanism underlying enzymatic PET degradation is the proximity effect,^17,21–24^ where active-site residues bring reactive groups into close contact, increasing their local concentration. For example, N-containing heterocycles (Lewis acid sites) in the enzyme interact with the carbonyl oxygen of PET, facilitating nucleophilic attack by hydroxyl groups and cleavage of ester bonds (Fig. 1b). While this mechanism offers valuable insights for synthetic catalyst design, directly mimicking the complex enzyme active sites is challenging due to their precise and complex structure. Fortunately, some metallohydrolases, especially binuclear metallohydrolases, also exhibit similar catalytic mechanisms *via* a proximity effects.^24^ To further simplify the structure of these hydrolytic enzymes, recently, we have demonstrated this proximity effect using a binuclear zinc synthetic catalyst to increase the local concentration of alkali and PET.^25,26^ However, two adjacent metal sites can easily hinder substrate adsorption and lead to metal leaching issues. To overcome this, we focused on other multifunctional Salen-based metal complexes, known for their structural tunability, stability, and biomimetic properties.^27–30^ These complexes, particularly those incorporating quaternary ammonium salts or organic bases, effectively accelerate reactions like epoxide ring-opening through synergistic interactions between the metal Lewis acid site and the nucleophilic base site.^29,31–34^ This mechanism, which enhances local concentration and stabilizes intermediates, suggests that Salen-based catalysts can potentially catalyze PET degradation *via* a proximity effect (Fig. 1c), offering a new, sustainable approach to plastic recycling.

To test this hypothesis (Fig. 1c), we synthesized a Robson-type Zn-Salen molecular catalyst with quaternary ammonium salt component through theoretical screening. Remarkably, the multifunctional Zn-Salen catalyst can efficiently depolymerize real-world post-consumer PET plastic wastes with high-crystallinity under mild conditions (*C*_OH^−^_ ≤ 0.1 M; *T* ≤ 90 °C). Mechanistic investigations indicate that the efficient PET depolymerization over this Zn-Salen catalyst originates from the synergistic effect of Zn metal site and quaternary ammonium component *via* a proximity effect, exactly matching the characteristics of PET hydrolases. This synthetic Zn-Salen catalyst remains robust over a wide range of operational temperature (30–350 °C) and pH (8–13). As a result, a productivity of 75 g_TPA_ L^−1^ h^−1^ was realized for sustainable PET recycling at *C*_OH^−^_ = 0.1 M and 90 °C. Besides, this synthetic catalyst is catalytically active toward a wide scope of substrates due to its high tolerance to various additives and impurities, including real-world waste PET products, PET-containing mixed plastics, and mainstream biodegradable PLA plastic. Techno-economic analysis shows that processing 100 thousand tons of waste PET plastic annually can generate a profit of 31 million USD and reduce greenhouse gas emissions by 230 thousand tons. This work will open up a new opportunity for the molecular design of artificial enzymes to better cope with the global waste plastic challenge.

## Results

### Theoretical calculation-assisted catalyst screening and characterization

**Fig. 2:**
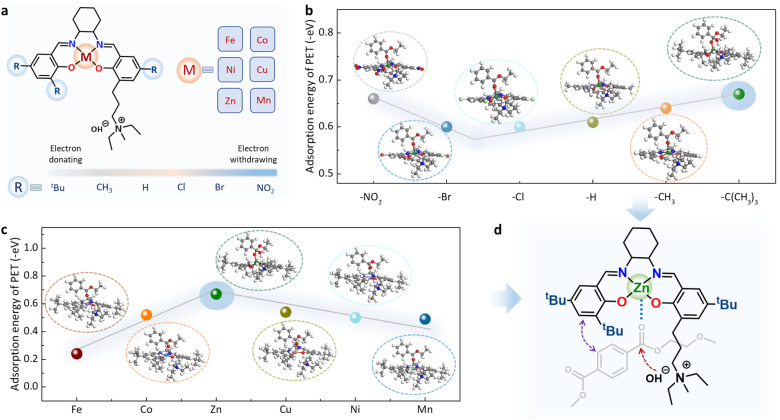
Theoretical calculation-assisted Salen-based molecular catalyst screening. (a) Structural design and regulation of Salen-based molecular catalyst. Adsorption energy of Salen-based molecular catalysts with different substituent functional groups (b) and metal centers (c) and on PET molecules. The inset depicts the model structures of PET molecules adsorbed on catalysts. (d) The optimal molecular structure of Zn-Salen with quaternary ammonium salt component.

To design a suitable Robson-type Salen molecular catalyst with a relatively high reaction activity, a key factor should be considered: a lower adsorption energy for PET molecules may be relatively advantageous for the activation and cleavage of the ester group in the PET molecule.^26,35^ Therefore, we first conducted density functional theory (DFT) calculations to screen a series of multifunctional Salen-based molecular catalysts with different metal centers (M = Fe, Co, Ni, Cu, Zn, Mn) and substituent functional groups (R = –NO_2_, –Br, –Cl, –H, –CH_3_, –^*t*^Bu) (Fig. 2a and S1). The relationship between adsorption energy and catalyst structure is shown in Fig. 2b and c. Generally speaking, metal Zn has the best catalytic activity in polyester hydrolysis reaction compared to other metal centers.^24–26^ Therefore, when examining different substituents, we preferentially chose metal Zn as the catalytic site. As shown in Fig. 2b, *tert*-butyl (–^*t*^Bu) as substituent is most favorable for the adsorption of PET molecule, which may be related to the stronger electron-donating capacity of –^*t*^Bu. The DFT results also indicate that the adsorption energy stability across metal centers follows the order: Zn (−0.67 eV) > Cu (−0.54 eV) > Co (−0.52 eV) > Ni (−0.50 eV) > Mn (−0.49 eV) > Fe (−0.24 eV) (Fig. 2c). This trend suggests that Zn-based catalysts exhibit the most stable adsorption, consistent with the Lewis acid strength of the metal ions. The stronger Lewis acidity of Zn enhances the binding and activation of the carbonyl groups in PET molecules. Based on these findings, the Zn-Salen molecular catalyst (Zn-Salen), with Zn as the metal center and –^*t*^Bu as the substituent, demonstrates the highest adsorption capacity for PET (Fig. 2d).

**Fig. 3:**
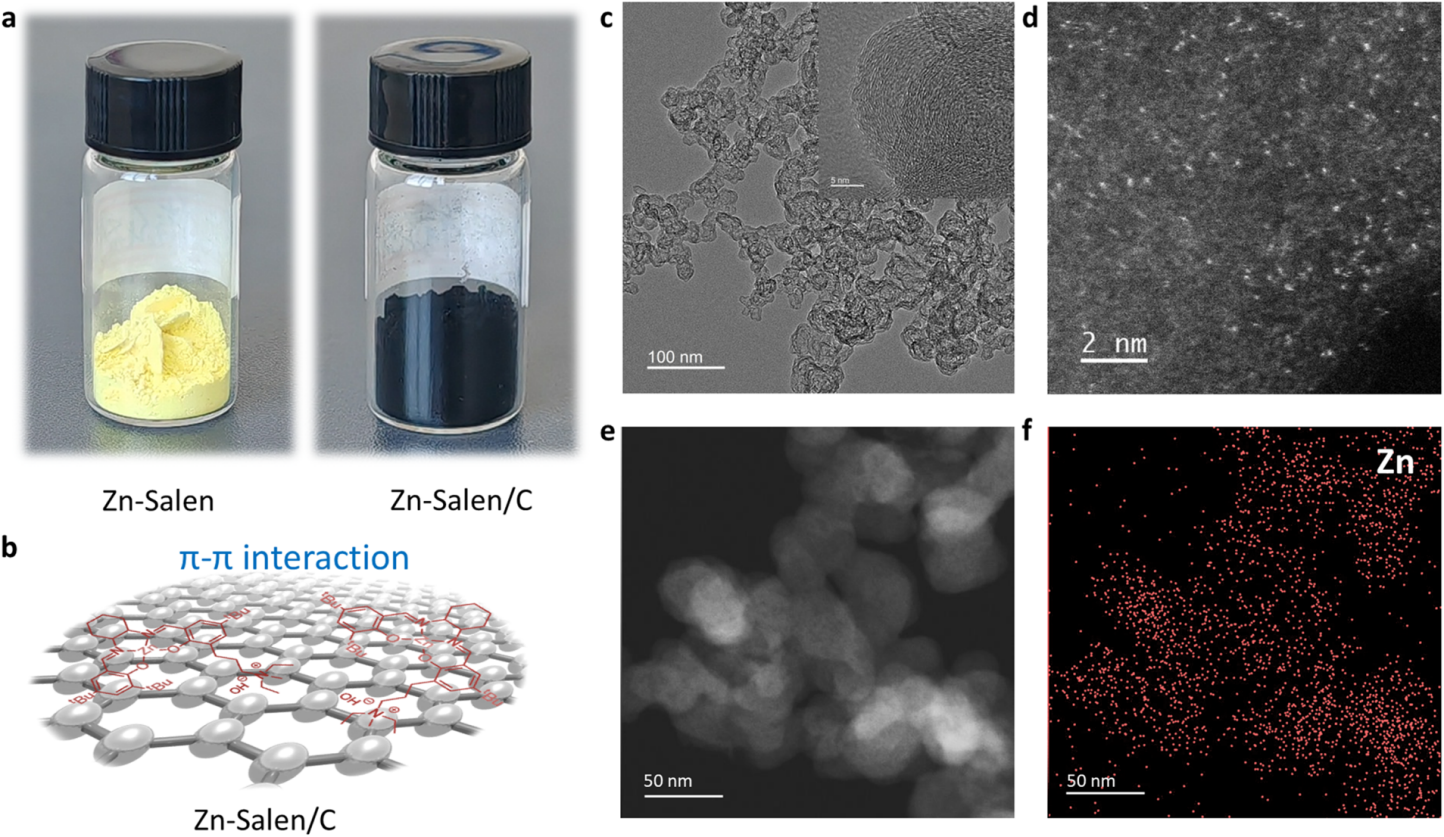
Zn-Salen/C catalyst characterization. (a) The photographs of Zn-Salen and carbon black nanoparticles (Ketjenblack EC-300J) supported Zn-Salen (Zn-Salen/C). (b) Structural model diagram of Zn-Salen adsorbed onto carbon black nanoparticles. (c) TEM and (d) the aberration-corrected HAADF-STEM of Zn-Salen/C. (e) HAADF-TEM and (f) mapping image of the element Zn on the Zn-Salen/C.

Guided by these theoretical insights, the optimal Robson-type Zn-Salen molecular catalyst with quaternary ammonium salt component was synthesized following established procedures (Fig. S2).^30,36^ For the convenience of subsequent catalyst separation and recycling, we further loaded Zn-Salen molecular catalyst on carbon black nanoparticles (Zn-Salen/C) based on the π–π interaction between the phenyl group in Zn-Salen and the sp^2^ carbon domain in the carbon black nanoparticles support (Fig. 3a and b). The transmission electron microscopy (TEM) images indicated that the material was composed of carbon black nanoparticles with size dimension of ∼30 nm (Fig. 3c). The high-resolution TEM (HR-TEM) image shows no obvious metal or metal oxide nanoparticles in the catalyst (Fig. 3c). More intuitively, aberration-corrected high-angle annular dark-field scanning transmission electron microscopy (AC-HAADF-STEM) image also shows that white bright spots (Fig. 3d) uniformly distributed on the carbon support, which can be assigned as single Zn sites. The energy dispersive X-ray spectrometry (EDS) mapping analysis reveals that Zn element is uniformly distributed on the carbon black nanoparticles (Fig. 3e and f). These above results indicate that the Zn-Salen retained molecular structural form without aggregation or decomposition.

**Fig. 4:**
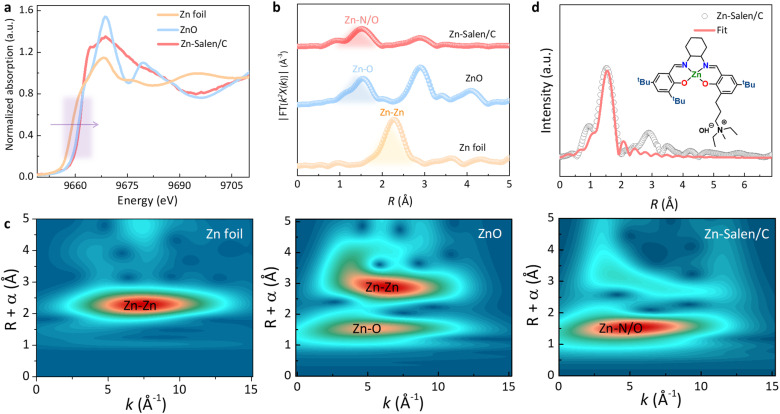
Fine-structure characterization of catalysts. (a) Normalized XANES spectra of Zn-Salen/C, and references. (b) Fourier transforms of EXAFS spectra of Zn-Salen/C, and references. (c) Wavelet transformed *k*^2^-weighted EXAFS for Zn foil, ZnO and Zn-Salen/C. (d) The structure model of ZnN_2_O_2_ motif based on which the profile of Zn-Salen/C is fitted.

The oxidation state and more precise local coordination environment of Zn sites in Zn-Salen/C were also investigated by X-ray absorption spectroscopy (XAS). X-ray absorption near-edge structure (XANES) spectroscopy revealed that the Zn K-edge of Zn-Salen/C shifted to higher energy compared to that of Zn foil and ZnO (Fig. 4a), suggesting a higher oxidation state of Zn sites in Zn-Salen/C than the references, which will favor the adsorption and activation of the ester carbonyl group in PET molecules. The coordination environment of the zinc sites in Zn-Salen/C was analyzed by Fourier transformed extended X-ray absorption fine structure (EXAFS). EXAFS shows that the zinc sites in Zn-Salen/C are coordinated to N/O atoms (1.5 Å) at the first coordination shell without forming Zn–Zn bond (2.3 Å) (Fig. 4b). The Zn–O/N coordination is more intuitively reflected from the wavelet transform (WT) analysis of the EXAFS data for Zn-Salen/C (Fig. 4c). The highest WT intensity is located in lobes centered at 4.5 Å^−1^ in *k*-space and 1.5 Å in *R*-space, corresponding to the N/O atoms around zinc site. Furthermore, the EXAFS spectrum matches well with the Zn-Salen molecular fitting model (Fig. 4d and S3), demonstrating that the Zn-Salen molecules retained their oxidation state and local coordination environment after being loaded onto carbon support.

### PET depolymerization under mild conditions

**Fig. 5:**
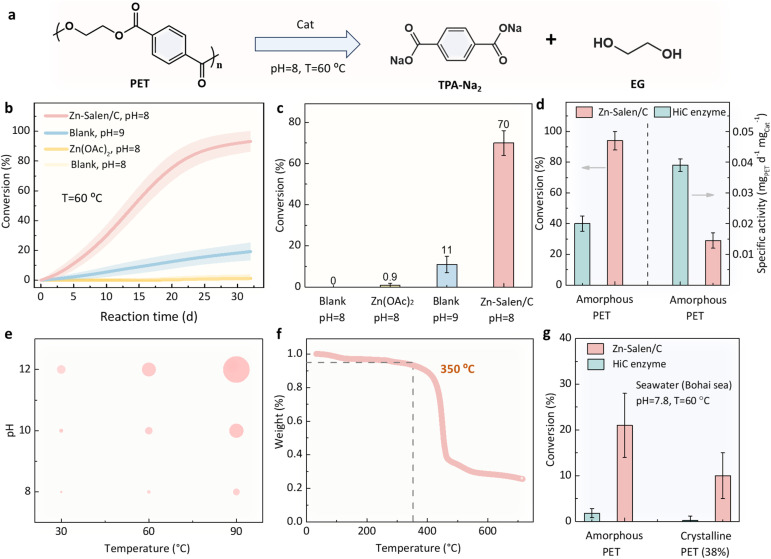
PET depolymerization over the Zn-Salen molecular catalyst. (a) PET hydrolysis process and products. (b) The hydrolysis kinetics of high-crystalline PET granule (38%) over Zn-Salen/C, Zn(OAc)_2_ and blank under pH 8 or pH 9 NaOH and 60 °C. (c) Conversion comparison over Zn-Salen/C, Zn(OAc)_2_ and blank within 18 days. (d) Conversion comparison for amorphous PET (am-PET, from Goodfellow) over Zn-Salen/C and commercially available *Humicola insolens cutinase* (HiC) under pH 8 and 60 °C. (e) The effects of pH and temperature on the specific activity of Zn-Salen/C, where the areas of the circles represent the magnitude of the specific activity. (f) Thermogravimetric analysis (TGA) of Zn-Salen, showing that the molecular catalyst was stable below 350 °C. (g) Conversion comparison for amorphous PET (am-PET, from Goodfellow) and high-crystalline PET granule (38%) over Zn-Salen/C and HiC in seawater (Bohai sea) with a pH 7.8 and 60 °C within 14 weeks.

The catalytic efficiency of the as-prepared Zn-Salen/C catalyst for hydrolyzing PET into monomers terephthalic acid (TPA) and ethylene glycol (EG) (Fig. 5a) was evaluated under mild conditions (pH 8, 60 °C) using high-crystalline PET (38%) as the substrate. As shown in Fig. 5b and c, the Zn-Salen/C catalyst achieved complete depolymerization of high-crystalline PET within 30 days. In comparison, both the blank experiment and the conventional hydrolysis catalyst, zinc acetate (Zn(OAc)_2_), exhibited negligible activity under the same conditions. Notably, traditional alkali hydrolysis typically requires harsher conditions, including higher alkali concentrations. A blank experiment at pH 9 further demonstrated that Zn-Salen/C at pH 8 exhibited sevenfold higher catalytic activity, underscoring its unique efficiency in breaking down chemically inert high-crystalline PET under mild conditions.

To further evaluate the performance of Zn-Salen/C, it was compared to the commercially available PET-degrading enzyme *Humicola insolens cutinase* (HiC). It should be pointed out that commercial HiC has relatively high degradation activity for polyester (not the most effective enzyme), but HiC was selected as the benchmark due to its wide availability in the laboratory and relatively heat-resistant and acid–base stable structure than PETases. The comparison was also conducted at pH 8 and 60 °C, an optimal condition for the commercial HiC. As shown in Fig. 5d and S4, HiC showed higher initial activity for amorphous PET (am-PET, from Goodfellow) with a specific activity of 0.038 mg_PET_ h^−1^ mg_catal_^−1^ than that of Zn-Salen/C. However, as the reaction time prolongs, HiC gradually becomes inactive (∼40% conversion), while Zn-Salen/C can achieve complete conversion of PET. Moreover, Zn-Salen/C exhibited comparable activity for high-crystalline PET, whereas HiC was nearly inactive for this substrate (Fig. 5b and S4). This highlights Zn-Salen/C's superior ability to degrade real-world, high-crystalline waste PET plastics.

Biological enzymes typically have strict operational requirements, such as specific temperature, pH, and ion concentrations, which limit their practical applications. To investigate the anti-interference ability of Zn-Salen catalyst to external environment, a wider range of temperature and pH parameters are adjusted for PET hydrolysis. Obviously, the Zn-Salen catalyst exhibits different degrees of catalytic activity over the entire temperature (30–90 °C) and pH (8–12) range, and the catalytic activity gradually increases with the increase of temperature and pH (Fig. 5e). Thermal stability tests (TGA) confirmed that Zn-Salen/C remains stable up to 350 °C (Fig. 5f). In addition, PET depolymerization was conducted in natural seawater to assess resistance to marine environmental interference, including complex salts. Zn-Salen/C exhibited catalytic activity an order of magnitude higher than commercial HiC (Fig. 5g), demonstrating superior anti-interference capability. These findings establish that the Zn-Salen molecular catalyst offers a broader operating range, higher stability, and greater practical application potential than existing PET-degrading enzymes.

### Real-world polyester plastic waste processing

**Fig. 6:**
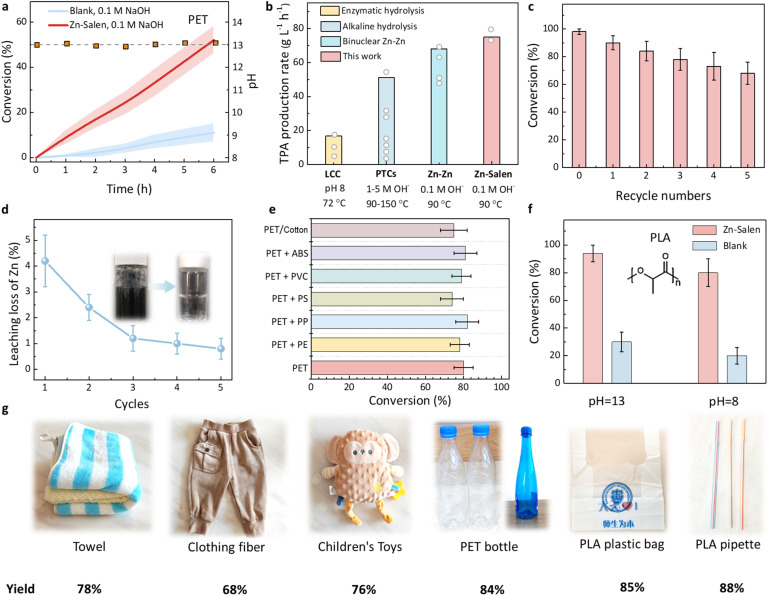
Real-world PET plastic waste recycling and wider applicability. (a) The hydrolysis kinetics of crystalline PET granule (38%) over Zn-Salen/C, and blank experiment (without catalyst) at pH 13 and 90 °C. (b) Space-time yield (STY) comparison for the reported enzyme-catalyzed PET hydrolysis, traditional alkaline hydrolysis with the presence of phase transfer catalysts (PTCs) and our previously reported binuclear zinc catalyst. (c) Recycling stability test of the Zn-Salen/C catalyst. (d) The percentage of zinc leaching for each cycle as determined by ICP-OES. Insets show the images of the solution after catalyst filtration. (e) Conversion comparison for PET-containing waste textiles (polyester/cotton), and PET-containing physical mixed plastics. (f) Conversion comparison for PLA plastic (Macklin, P909229, Mn ≈ 80 000, crystallinity 25%, 100 mesh) over Zn-Salen/C, and blank experiment (without catalyst) at pH 8 or pH 13 and 60 °C. (g) Catalytic activity of real-world post-consumer PET and PLA products with high crystallinity and complex mixtures over Zn-Salen/C. All the error bars in this figure represent the standard deviations for three measurements.

Given the broad operational range of the Zn-Salen/C catalyst, we investigated its performance in scalable PET recycling at pH 13 and 90 °C. As shown in Fig. 6a, Zn-Salen/C achieved a PET conversion rate five times higher than the blank experiment (without catalyst). The catalyst delivered an industrially relevant space-time yield (STY) of 75 g_TPA_ L^−1^ h^−1^ (Fig. 6b), outperforming other hydrolysis processes, including enzyme-catalyzed PET hydrolysis (maximum STY: 16.7 g_TPA_ L^−1^ h^−1^), our previously reported binuclear zinc catalyst (50–70 g_TPA_ L^−1^ h^−1^),^25^ and traditional alkaline hydrolysis (maximum STY: 51.3 g_TPA_ L^−1^ h^−1^), which typically requires 90–150 °C in 1–5 M alkaline solutions with phase transfer catalysts (PTCs) (Fig. 6b). Catalyst reusability, a critical factor for industrial applications, was also assessed. Zn-Salen/C exhibited a ∼30% decrease in yield after five reuse cycles (Fig. 6c), primarily due to catalyst mass loss and Zn leaching during filtration recovery. Inductively coupled plasma optical emission spectroscopy (ICP-OES) revealed a cumulative Zn loss of 9.6% over five cycles (Fig. 6d), which corresponded to the activity reduction. Future work should focus on optimizing the catalyst structure to enhance stability and minimize leaching. Nevertheless, AC-HAADF-STEM and wavelet transform analysis of the EXAFS data for Zn-Salen/C after reaction show that the remaining zinc is still uniformly distributed on the carbon carrier in the atomic state (Fig. S5).

As a further step towards real-world applications, we submitted the Zn-Salen/C catalyst to different PET feedstocks. As illustrated in Fig. 6e, the conversion of PET components all exceeded 70% for PET-containing waste textiles (polyester/cotton clothing), and PET-containing physical mixed plastics including polyvinylchloride (PVC), polystyrene (PS), polypropylene (PP), acrylonitrile butadiene styrene (ABS) plastic, and polyethylene (PE). These results highlight the catalyst's capability to process real-world mixed plastic waste effectively. In addition, Zn-Salen/C exhibited high catalytic activity for biodegradable polylactic acid (PLA) plastics (Fig. 6f). This versatility contrasts with the specificity limitations of plastic-degrading enzymes, which are often ineffective for substrates beyond PET.^37–39^ Finally, the catalyst was applied to post-consumer PET and PLA products with high crystallinity and complex compositions, such as dyed bottles, clothing fibers, children's toys, towels, PLA bags, and PLA pipettes. Conversion rates for these materials ranged from 68% to 88% (Fig. 6g), demonstrating the Zn-Salen/C system's robustness and anti-interference capability for real-world polyester waste recycling.

### Closed-loop PET recycling

**Fig. 7:**
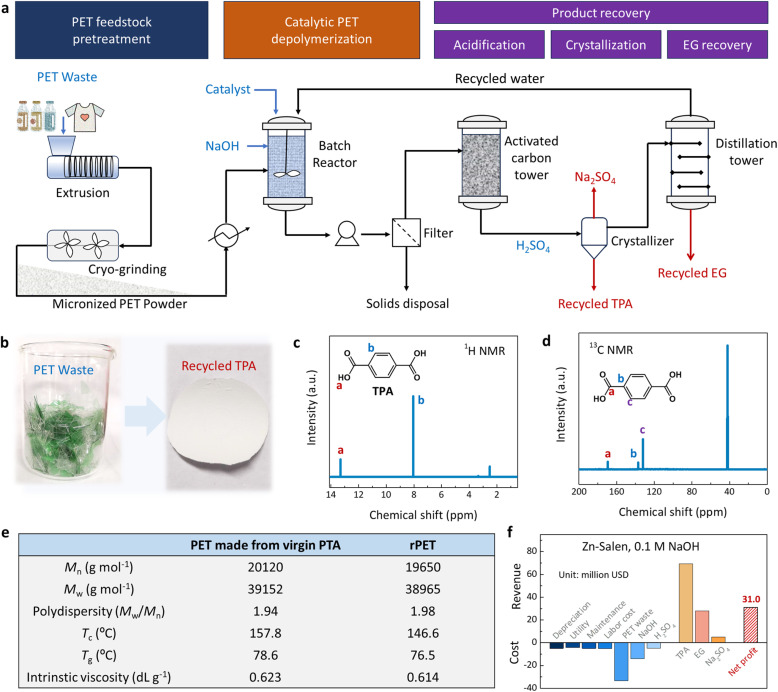
PET recycling process, techno-economic analysis. (a) Simplified flow diagram of the PET recycling process over Zn-Salen/C. (b) TPA monomers obtained by recycling. (c) ^1^H NMR spectrum and (d) ^13^C NMR spectrum of recovered TPA. (e) Comparison of rPET made from the recycled PTA with the PET made from virgin PTA. (f) Techno-economic analysis of processing 100 thousand tons of waste PET annually based on this biomimetic catalytic system.

To demonstrate a closed-loop PET recycling, we also recovered TPA monomer product from post-consumer waste PET bottle with a 50 g handling capacity (solids content of 16.7 wt% and PET/catalyst ratio of 500 g_PET_ g_catal_^−1^) at pH 13 and 90 °C. After the reaction, catalyst was recycled for reuse by vacuum filtration. Then, pure terephthalic acid (TPA) was obtained by acidification, filtration, washing and drying process (Fig. 7a). Test analysis found that the recovered TPA (Fig. 7b) have a high purity as confirmed by ^1^H and ^13^C nuclear magnetic resonance (NMR) and FT-IR spectrum (Fig. 7c, d and S6, S7). However, according to TPA quality testing (Table S1), the purity of recycled TPA still needs to be improved (such as multiple washings, adsorption, *etc.*) to meet bottle-grade standards. Going one step further, the recovered TPA was used as a raw material to make recycled PET (rPET). The parameter characteristics of the rPET shows that rPET was similar to those of PET made from virgin PTA (Fig. 7e). Based on this biomimetic catalytic system, we also performed a tentative techno-economic analysis (TEA) to evaluate the market profit and loss situation. The processing costs mainly include equipment depreciation, utilities, labor, maintenance, PET waste, alkali and acid, and the revenue comes mainly from TPA, EG and Na_2_SO_4_. TEA shows that processing 100 thousand tons of PET plastic waste per year could generate a net profit of US$31.0 million (Fig. 7f and Table S2), significantly higher than the traditional alkaline hydrolysis process at 1.0 M NaOH (US$8.2 million) with the presence of phase transfer catalysts (PTCs) (Fig. S8 and Table S3), which indicates that this biomimetic catalytic system has potential commercial value.

Furthermore, the positive environmental impact of this biomimetic catalytic system was also evaluated. Calculations estimated that if 100 thousand tons of PET waste were landfilled or incinerated, approximately 230 thousand tons of CO_2_ will be emitted into the atmosphere each year, which will accelerate the global “greenhouse effect”. In marked contrast, using this biomimetic catalytic system not only has the potential to generate significant commercial value, but also leads to a significant reduction in CO_2_ emissions (0 tons *vs.* 230 thousand tons). These results indicate that this biomimetic catalytic system could significantly reduce carbon emissions and have potential commercial value.

### Catalytic mechanism

**Fig. 8:**
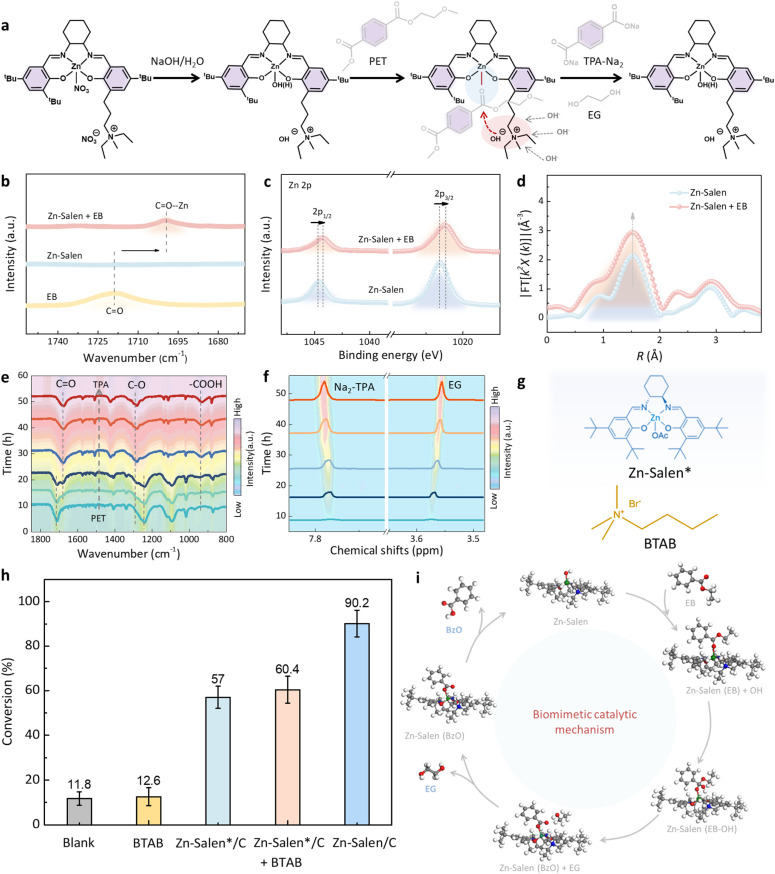
PET depolymerization mechanism over the Zn-Salen molecular catalyst. (a) The key steps during the PET hydrolysis over Zn-Salen catalyst. (b) DRIFTS spectra of ethyl benzoate (EB), an analogue to PET but with low boiling point, over the Zn-Salen molecular catalyst. (c) XPS spectra and (d) Fourier transforms of EXAFS spectra of Zn-Salen catalyst with or without EB. (e) FT-IR spectra and (f) ^1^H NMR analysis during the PET depolymerization under pH 13 and 60 °C (to accelerate this reaction). (g) Molecular structure of Robson-type Zn-Salen molecular catalyst without quaternary ammonium salt component (Zn-Salen*) and molecular structure of pure quaternary ammonium salt (butyltrimethylammonium bromide, BTAB). (h) Conversion comparison for PET over Zn-Salen/C, Zn-Salen*/C, Zn-Salen*/C + BTAB, BTAB and blank at pH 13 and 60 °C. (i) The proposed PET biomimetic catalytic mechanism over Zn-Salen.

To explore the unique catalytic activity of this synthetic catalyst, we first proposed a possible biomimetic catalytic process based on the enzymatic PET depolymerization mechanism. As shown in Fig. 8a, some key steps are involved during PET depolymerization, mainly including catalyst adsorption on the PET plastic surface, metal Zn sites coordinating with O atom in the C=O group of PET molecule and activating C=O bond, nucleophilic groups attacking the C atom in the C=O group and promoting C–O bond cleavage, and desorption of monomer products.

To verify the correctness of this mechanism, we first investigated the adsorption of carbonyl group using diffuse reflection infrared Fourier transform spectroscopy (DRIFTS). With the presence of the Zn-Salen, the carbonyl stretch presented a red shift from 1719 cm^−1^ to 1699 cm^−1^ (Fig. 8b). It suggests a bond-weakening effect on the carbonyl group upon coordination to Zn site, whereby the electrophilicity of the substrate is enhanced,^40–43^ which is consistent with the results of XPS (Fig. 8c). EXAFS revealed that the coordination number of Zn did increase when ester was introduced (Fig. 8d), further confirming the coordination between ester carbon group and metal Zn site.

To demonstrate the cleavage of C–O bonds and the desorption behavior of monomer products, Fourier transformed infrared (FT-IR) and liquid ^1^H NMR were performed during the PET depolymerization. As shown in Fig. 8e, the C=O groups and C–O groups of PET plastic gradually disappeared, while C=O, C–O and –COOH groups of TPA monomer gradually appeared with increasing reaction time, indicating the cleavage of C–O bonds and the generation of monomers. Further liquid ^1^H NMR analysis showed that two peaks at ∼7.78 and ∼3.57 corresponded to monomer TPA-2Na and EG (Fig. 8f), respectively, and the areas of the two peaks increased significantly with increasing depolymerization time. These results indicate that the monomer products immediately desorbed from the PET plastic surface or on catalytic site and entered the solution after depolymerization.

To gain further insights into the synergistic effect of the Zn site and quaternary ammonium component in Zn-Salen molecular catalyst during PET depolymerization, we also performed a series of control experiments and density functional theory (DFT) calculations. As shown in the Fig. 8g, h and S9 control experiments with various catalyst configurations (Zn-Salen* without the quaternary ammonium component, pure quaternary ammonium salt (butyltrimethylammonium bromide, BTAB), and Zn-Salen* + BTAB physically mixed components) show that Zn-Salen molecular catalyst with the quaternary ammonium component exhibits significantly high catalytic activity, confirming that the synergistic interaction between Zn sites and the quaternary ammonium component is critical for nucleophilic activation. Potential energy calculations demonstrated that the Zn site stably binds to the carbonyl group of PET (Fig. S10). The proximity effect of the quaternary ammonium component further enhances nucleophilic attack by hydroxyl groups, promoting C–O bond cleavage. After C–O bond cleavage, the active site becomes temporarily coordinated by the resulting carboxylate and is restored *via* ligand exchange with hydroxide. DFT calculations also revealed that monomer desorption is the most endothermic step, with energy barriers of 0.57 eV for TPA-2Na and 0.84 eV for EG. Whereas in the actual reaction, due to the presence of temperature and stirring dynamics, the monomer products with high water solubility are very susceptible to desorption from the PET plastic surface or on catalytic site into aqueous solution, as confirmed by the FT-IR and ^1^H NMR results (Fig. 8e and f). The calculated potential energy profiles indicate that the whole process is energetically feasible under the reaction conditions. These results also indicate that the synergistic effect of Zn metal site and quaternary ammonium component in Zn-Salen enhance adsorption, activation and nucleophilic attack processes towards the O–C=O groups in PET molecule *via* a proximity effect (Fig. 8i), mimicking the functional characteristics of PET hydrolases.^17,21–24^

## Discussion

In summary, we designed a Zn-Salen molecular catalyst for polyester depolymerization. Mechanistic studies reveal that the proximity effect between Zn metal site and quaternary ammonium component increases the local substrate concentration at the active site and accelerates the adsorption, activation and nucleophilic attack processes towards the O–C=O group in PET, exactly matching the characteristics of PET hydrolases. Due to high resistance to external interference, this synthetic catalyst provides an industrially feasible productivity of 75 g_TPA_ L^−1^ h^−1^ for centralized PET recycling at *C*_OH^−^_ = 0.1 M and *T* = 90 °C. We further demonstrated the compatibility of this synthetic catalyst with complex mixtures in plastic waste and its wide substrate scope. Techno-economic analysis and environmental assessment shows that this biomimetic catalytic system can significantly increase net profits and reduce carbon emissions. Considering that the structure of the reported Zn-Salen complex can be further tailored in many ways, future work should focus on the structure design of enzyme-mimicking catalyst to improve structural stability. For example, changing the coordination environment around the metal Zn site can enhance its binding ability and prevent Zn loss. Alternatively, homogeneous Zn-Salen complexes can be polymerized into heterogeneous polymers or covalent organic frameworks (COFs), which not only have a structure closer to that of enzymes but also facilitate better catalyst recovery. We expect that this work will be the starting point for the development of more efficient enzyme-mimicking catalysts for plastic recycling.

## Author contributions

S. Z. conceptualized and guided this work. S. Z. and M. L. designed the experiments. M. L. and L. T. performed the experiments. Y. S. and N. J. performed the DFT calculations. S. Z., M. L. and L. T. wrote the paper. All the authors participated in the data analysis and commented on the manuscript.

## Conflicts of interest

The authors declare no conflict of interest.

## Supplementary Material

SC-016-D5SC04667J-s001

## Data Availability

Supplementary information: The data that support the findings of this study are available within the article and SI. See DOI: https://doi.org/10.1039/d5sc04667j.
