# Supplementary material for: Theory-guided multifunctional Zn-Salen molecular catalyst for sustainable polyester plastic recycling
Source: Chem Sci. 2025 Aug 25;16(37):17334–44. doi: 10.1039/d5sc04667j (PMC12400447; doi:10.1039/d5sc04667j)
Supplement: SC-016-D5SC04667J-s001 [file SC-016-D5SC04667J-s001.pdf]

Supplementary Information (SI) for Chemical Science.
This journal is © The Royal Society of Chemistry 2025

Supporting Information

Theory-guided multifunctional Zn-Salen molecular catalyst for sustainable polyester plastic recycling

Mei Li[1,2,4] Yawen Shi,[1,4] Lei Tang,[3,*] Na Ji,[1,*] & Shengbo Zhang[1,*]

[1]School of Environmental Science and Engineering, Tianjin Key Laboratory of Biomass/Wastes Utilization, Tianjin University, Tianjin 300350, China

[2]School of Materials Science and Engineering, Smart Sensing Interdisciplinary Science Center, Nankai University, Tianjin 300350, China

[3]Institute of Sustainability for Chemicals, Energy and Environment (ISCE2), Agency for Science, Technology and Research (A*STAR), 1 Pesek Road, Singapore, 627833 Republic of Singapore

[4]These authors contributed equally: Mei Li, Yawen Shi

[*]E-mail: shengbozhang@tju.edu.cn; [*]E-mail: jina@tju.edu.cn; *Email: Tang_Lei@isce2.a-star.edu.sg

Table of Contents

1. Materials and methods

2. Techno-economic analysis

3. Figures S1-10

4. Tables S1-3

5. References

1. Materials and methods

Syntheses of the catalysts

Robson-type Zn-Salen molecular catalyst with quaternary ammonium salt component (Zn-Salen) was prepared according to procedures previously reported with minor modifications[1,2]. The obtained compounds were characterized by ^{1}H NMR. ^{1}H NMR (400 MHz, $CDCl_3$):13.70 (s, 1H), 13.50 (s, 1H), 8.34 (s, 1H), 8.30 (s, 1H), 7.32 (s, 1H), 7.16 (s, 1H), 7.06(s, 1H), 7.01(s, 1H), 3.10-3.40 (m, 8H), 2.97(s. 3H), 2.69(m, 2H), 1.50-2.00 (m, 10H), 1.38 (s, 9H), 1.20-1.30 (m, 24H). Robson-type Zn-Salen molecular catalyst without quaternary ammonium salt component (Zn-Salen*) was purchased in Bidepharm (556053-31-7, 95%, 250 mg) and were characterized by ^{1}H NMR and FT-IR (**Fig. S9**). For syntheses of the Zn-Salen/C, Carbon support (Ketjenblack EC-300J) was dispersed in methanol/water mixture ($V_{methanol}/V_{water}$ = 1/1). The dispersion was ultrasonicated for 10 min. Meanwhile, Zn-Salen was dissolved in another methanol/water mixture ($V_{methanol}/V_{water}$ = 1/1). Then, Zn-Salen was added into the carbon-containing solution and was ultrasonicated for 10 minutes. Finally, the solid product was collected by filtration and dried in vacuum oven. The content of the metal Zn is 2.7 wt% based on ICP-OES and elemental analysis.

PET hydrolysis

PET hydrolysis was performed in a 20 mL vial placed in an aluminum heating block on a stirring hotplate. Zn-Salen/C catalyst (0.8 mg of zinc),10 mg of crystalline PET granule (Macklin, P875573, crystallinity of 38%, micronized into 100 mesh) or amorphous PET film (Goodfellow, ES301445, micronized into 100 mesh), and 10 mL of NaOH solution (pH 8 or pH 9) or natural seawater (Bohai sea, China, pH 7.8) were added into the vial. The temperature of the hydrolysis reaction was set at 60 ºC, and the pH of the solution was maintained by adding NaOH. For PET hydrolysis over HiC was conducted in potassium phosphate buffer (pH 8, 1.0 M). For optimized large-scale PET hydrolysis experiments, 50 g of PET powders, and 50 mg Zn-Salen supported on carbon, 50 mL of NaOH solution (pH 13) were added into a 250 mL three-necked flask under stirring. The reaction temperature was set at 90 ºC, and the pH value of the solution was maintained constant by adding NaOH. For closed-loop PET recycling experiment, post-consumer PET water bottles were pretreated with liquid nitrogen and then micronized into powders with diameters of 100–500 μm using a crusher. After hydrolysis under reaction conditions (pH 13. 90 ºC), the catalyst was filtered out, acid was added to the filtrate to convert TPA-2Na to white precipitated TPA, and pure solid TPA was obtained by filtration, washing and drying.

Characterization

Zn K-edge X-ray absorption spectra (XAS) analysis was performed at the 4B9A station in Beijing Synchrotron Radiation Facility (BSRF), operated at 2.5 GeV with a maximum current of 250 Ma with Zn foil, and ZnO were used as reference materials. Aberration-corrected high-angle annular dark-field scanning transmission electron microscopy (AC-HAADF-STEM) measurements were conducted using a JEOL JEM-ARM200F instrument. Diffuse reflection infrared Fourier transform spectroscopy (DRIFTS) was obtained via a Fourier transform infrared spectrometer (Thermo Nicolet iS50) equipped with a diffuse reflection accessory (Harrick). X-ray photoelectron spectroscopy (XPS) was conducted on a Thermo Scientific ESCALAB 250Xi. Fourier Transform Infrared Spectroscopy (FT-IR) was conducted using a Nicolet Magna 670 FTIR spectrometer. ^{1}H NMR spectra were measured on 800 MHz Bruker spectrometer (AVANCE NEO 800) with D_2O as solvent and maleic acid (MA) as the internal standard for PET hydrolysis. The quantitative analysis is based on the following equation[3,4]:

$$n_{analyte} = \frac{S_{analyte}}{S_{standard}} \times \frac{2}{N_{monomer}} \times \frac{m_{standard}}{M_{standard}} \times \frac{10}{0.1}$$

The conversion of PET (%)

$$conversion = \frac{n_{analyte}}{m_{PET}} \times M_{unit} \times 100\%$$

Where $S_{analyte}$ and $S_{standard}$ represent the integral areas of peaks for MA and the analyte, respectively. Where $n_{analyte}$ represent the molar quantity of the analyte. The $N_{monomer}$ represents the number of hydrogen atoms in the characteristic peak of the monomer used for quantitative analysis. $m_{standard}$ and $M_{standard}$ represent the mass and mole weight of MA, respectively. m_{PET} represents the initial mass of PET. M_{unit} represents the molecular weight of the repeating unit ($C_{10}H_8O_4$) of PET.

DFT calculations

Spin-polarized periodic DFT calculations were performed by the Vienna ab initio simulation package (VASP, version 5.4.4).[5,6] The projector augmented wave (PAW)[7,8] was used to describe electron-ion interactions. The Perdew Burke Ernzerhof (PBE) functional[9] was employed to calculate electron exchange and correlation energies, and the Grimme's DFT-D3[10] method was added to study the effect of van der Waals interaction. All DFT calculations were performed at a cut-off energy of 400 eV. The convergence criteria for the electronic self-consistent convergence and force were set to 10^{-4} eV and 0.05 eV/Å. To capture the intermediates, the model was constructed within a vacuum cube of 30 × 30 × 30 Å.

2. Techno-economic analysis

The cost includes maintenance cost, equipment depreciation cost, utility cost, labor cost and chemical cost.

Maintenance cost: Maintenance cost refers to the expense incurred for the upkeep of facilities, which is typically estimated to be 10% of the capital cost.

Equipment depreciation cost: This variable represents the capital cost in relation to its service life. In this context, we have assumed that the salvage value would be zero after a service life of ten years.

Utility cost: Utility cost encompasses the expenses related to power, steam, and compressed air. Among the various processes, evaporation, distillation, and crystallization represent the most significant costs. The annual capacity for PET treatment is established at 100000 tons and 70000 tons for Zn-Salen/C and traditional alkaline hydrolysis, respectively, based on different space-time yield (STY). It can be roughly estimated that the remaining utility costs are comparable to those of evaporation and distillation. The evaporation cost was determined based on the price of an equivalent amount of steam, with the steam price provided by the DOE benchmark.

Labor cost: Labor cost pertains to the expenses associated with employing workers. It was estimated that labor costs would amount to 10% of the capital cost.

Chemical cost: Table S2−3 display the prices of the chemicals. All data was obtained from a chemical trading platform named SunSirs (http://www.sunsirs.com/) and verified on another platform, made-in-china (https://www.made-in-china.com/), in 2021.

3. Figures S1-10

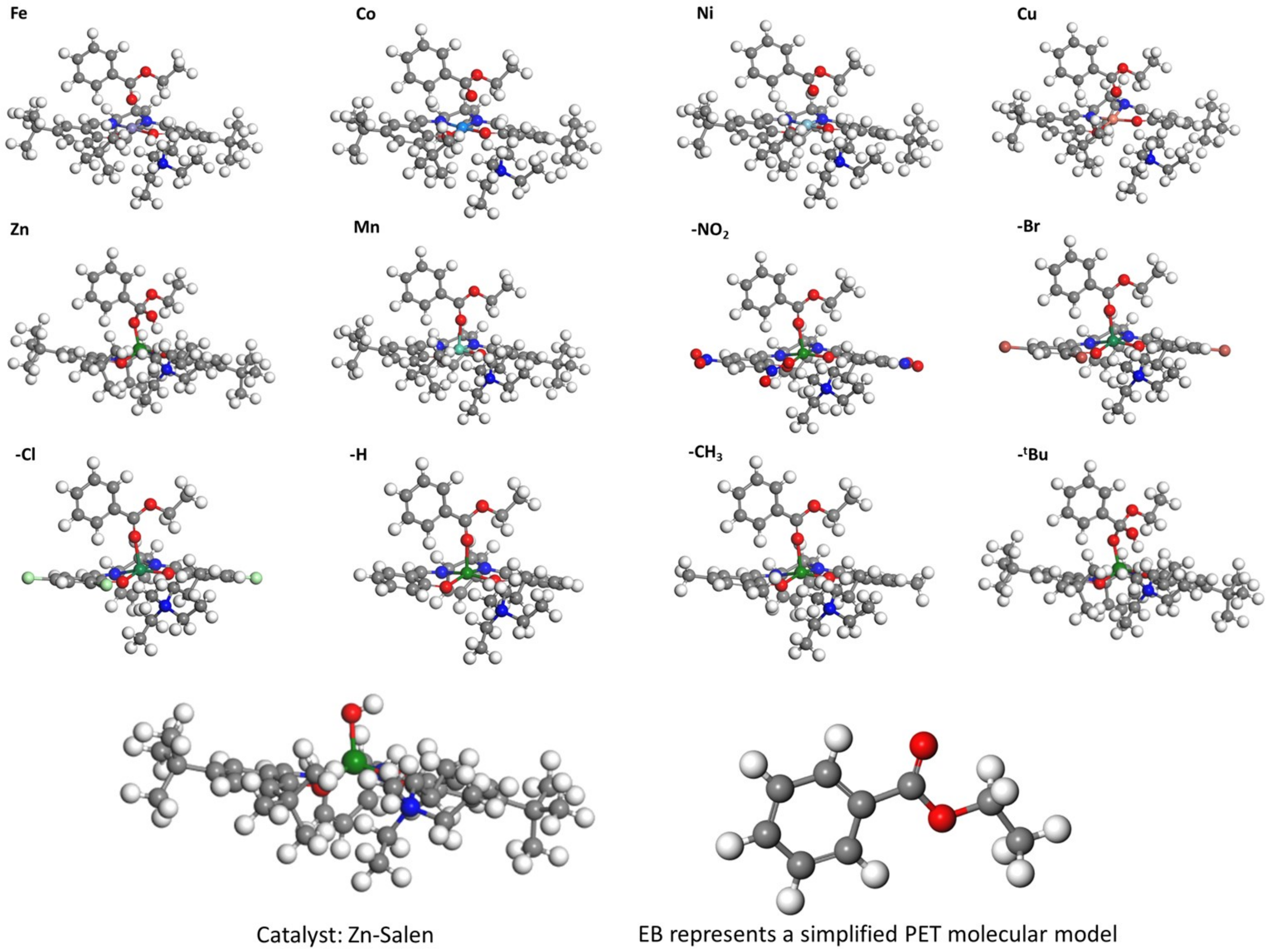

Figure S1. The adsorption configurations of PET on Salen-based molecular catalysts with different metal centers (M = Fe, Co, Ni, Cu, Zn, Mn) and substituent functional groups (R= –NO_2, –Br, –Cl, –H, –CH_3, –^{t}Bu).

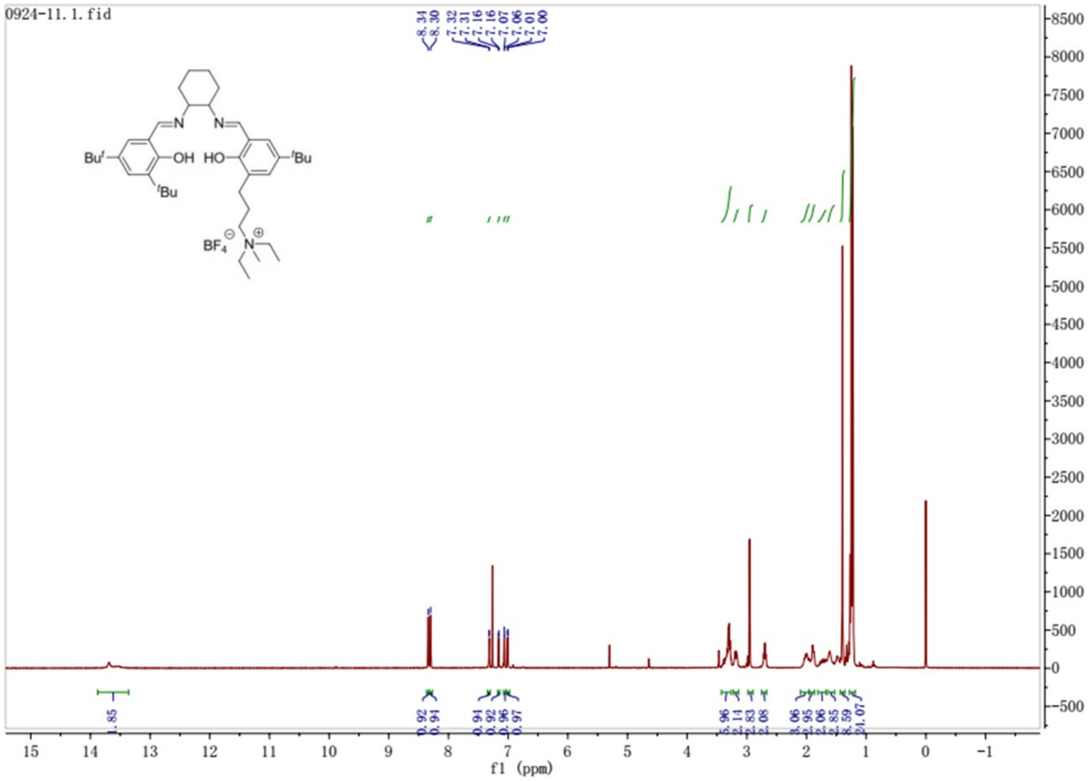

Figure S2. ^{1}H NMR spectrum of Salen with quaternary ammonium salt component and –^{t}Bu functional group.

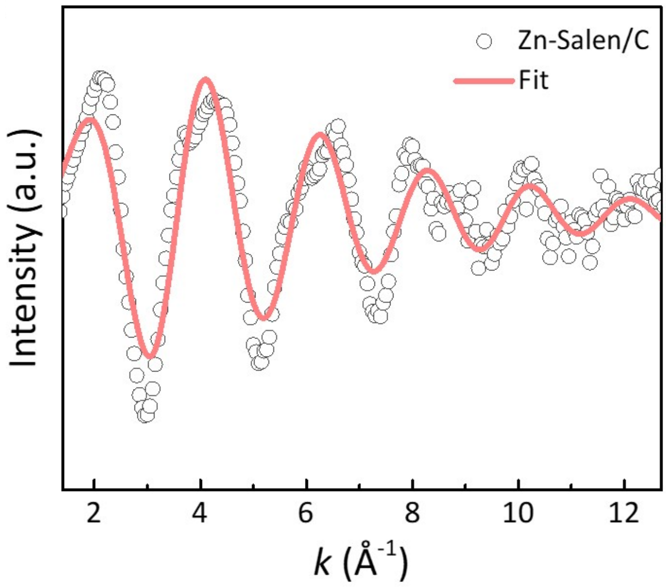

Figure S3. The EXAFS χ(k) data at Zn *K*-edge for Zn-Salen/C and the fitting based on ZnN_2O_2 structure model.

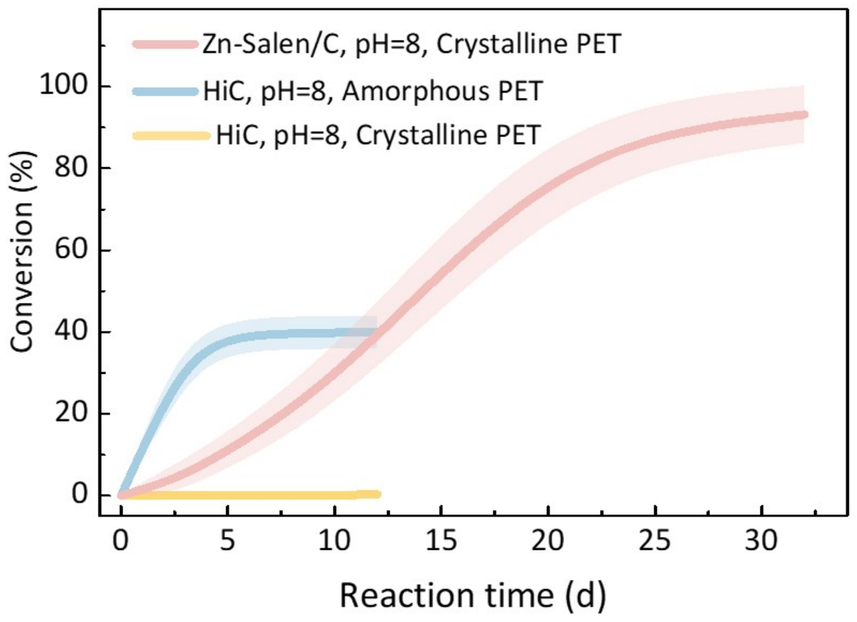

Figure S4. The hydrolysis kinetics of high-crystalline PET granule (38%) and amorphous PET over Zn-Salen/C and HiC.

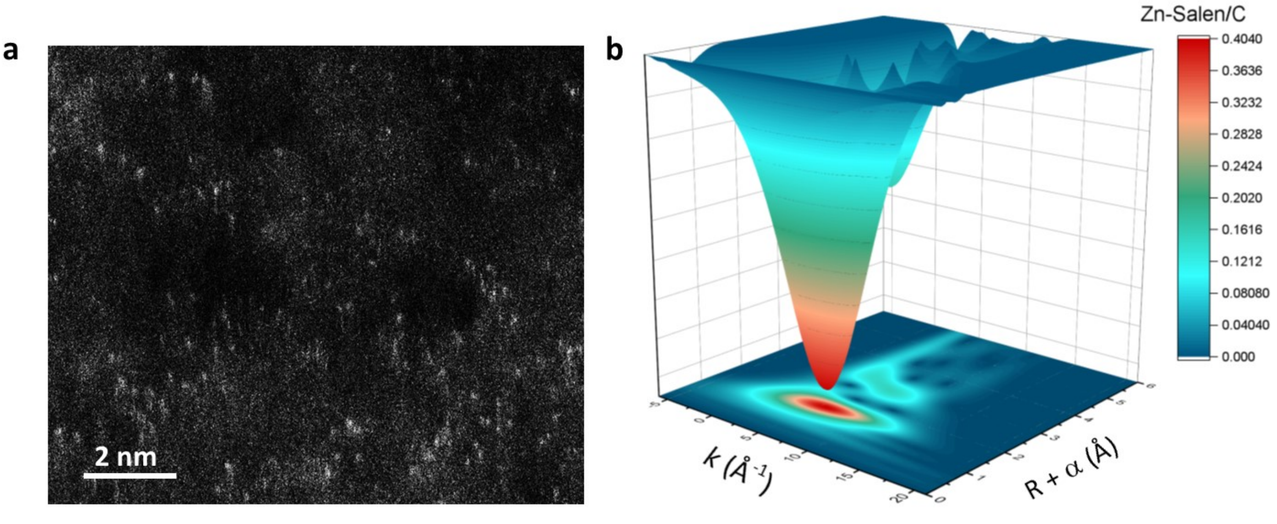

Figure S5. (a) The aberration-corrected HAADF-STEM and (b) Wavelet transformed k^2-weighted EXAFS of Zn-Salen/C after reaction.

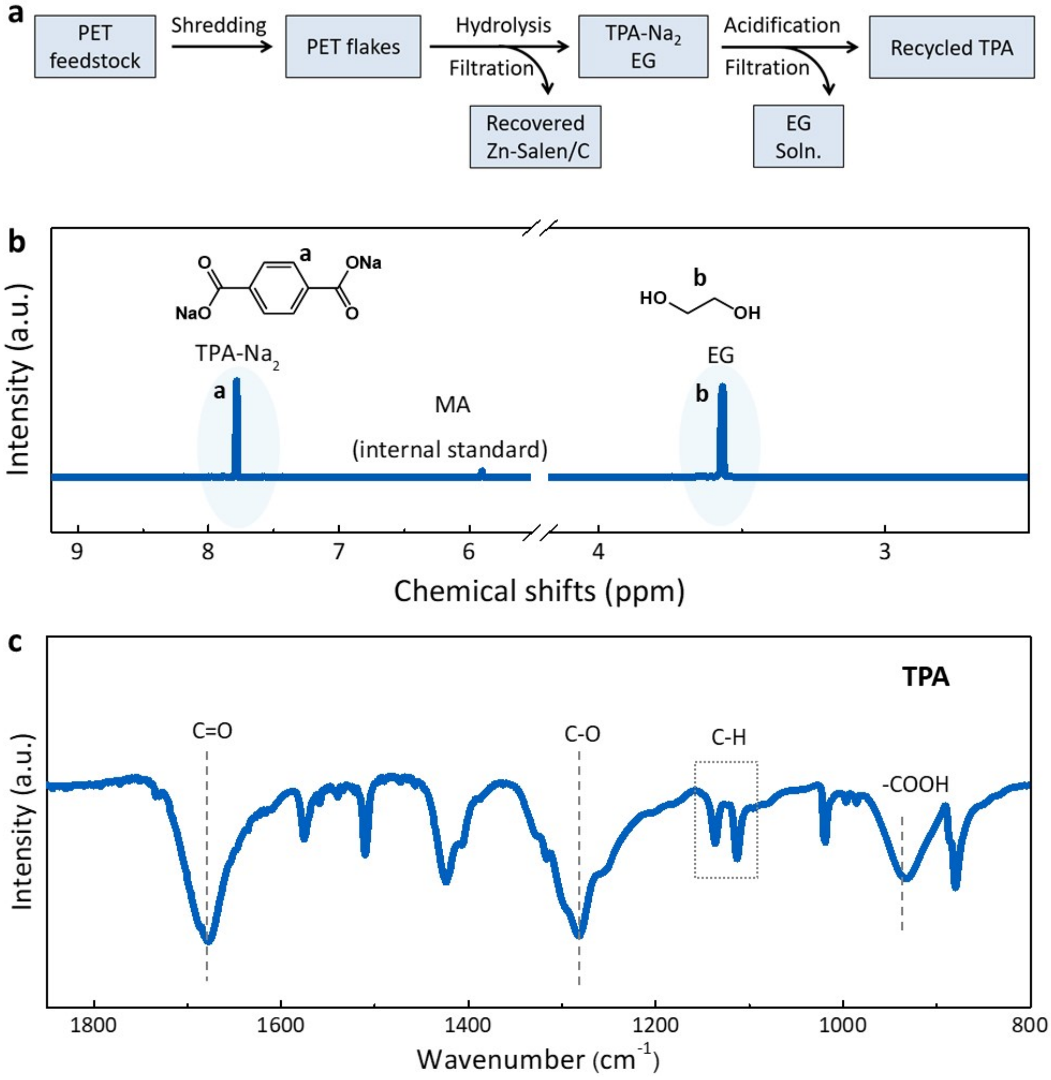

Figure S6. (a) The flow diagram of the PET recycling process. (b) ^{1}H NMR spectrum of recovered TPA and EG. (c) FT-IR spectrum of recovered TPA.

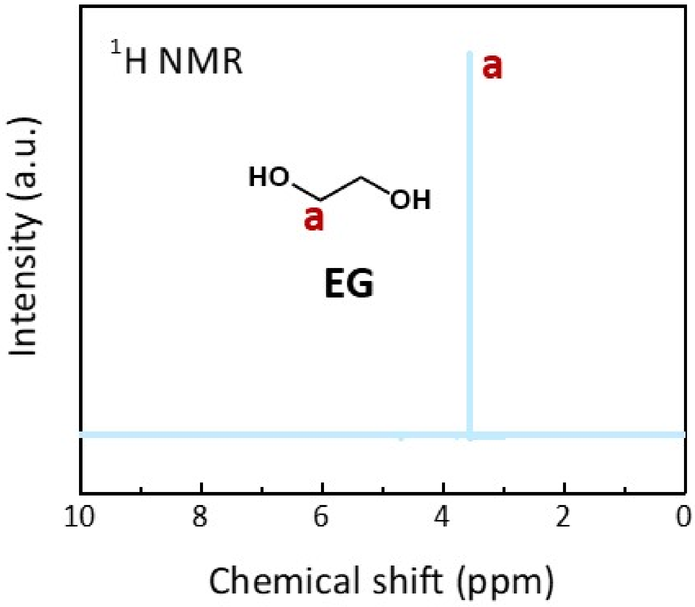

Figure S7. ^{1}H NMR spectrum of EG.

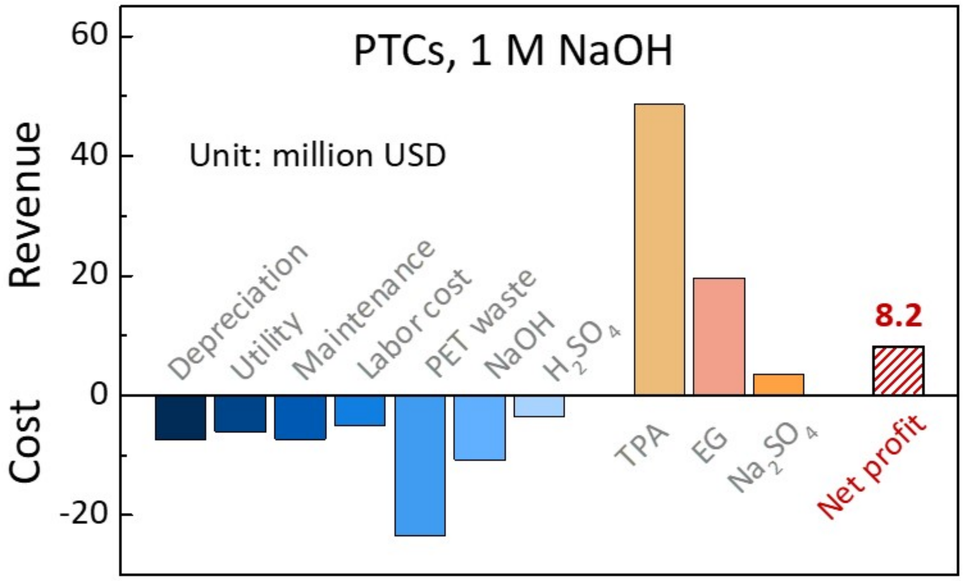

Figure S8. Techno-economic analysis of processing 70 thousand tons of waste PET annually based on a traditional hydrolysis process (1.0 M NaOH) with the presence of phase transfer catalysts (PTCs).

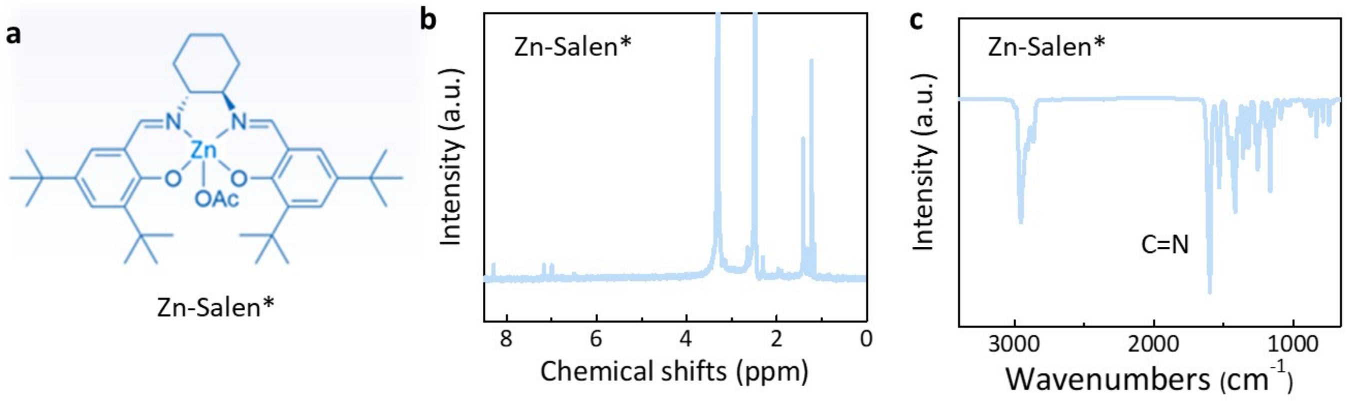

Figure S9. (a) Molecular structure of Robson-type Zn-Salen molecular catalyst without quaternary ammonium salt component (Zn-Salen*). (b) ^{1}H NMR spectrum and (d) FT-IR spectrum of Zn-Salen*.

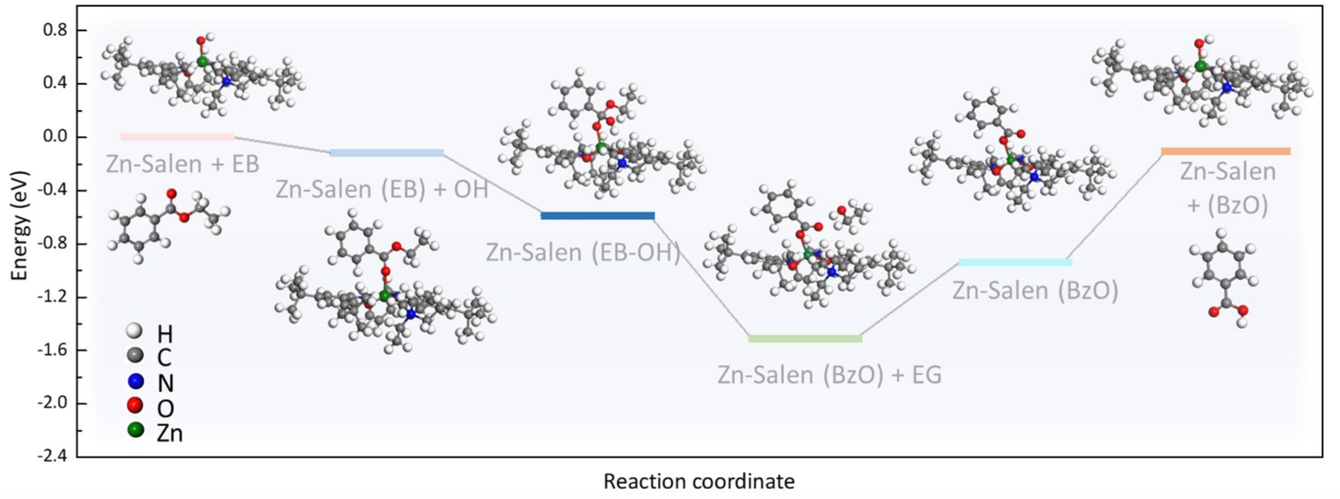

Figure S10. Density functional theory (DFT) calculations. Potential energy profile of PET depolymerization on Zn-Salen catalyst. The inset shows the DFT optimized geometries for reactants and intermediates.

4. Tables S1-3

Table S1. Quality testing of recycled TPA.

	Bottle-grade indicators	rTPA
T340, %	≥90	83.2
T400, %	≥97	92.7
Total heavy metal content (Ni, Co, Ti, Mn), mg/kg	≤3	5.2
Fe, mg/kg	≤1	0.15
Na, mg/kg	≤0.6	3.2
3g/100ml DMF chroma	≤10	10

Table S2. Simplified techno-economic analysis based on a capability of 100000 tons of PET waste per year for alkaline hydrolysis in 0.1 M NaOH catalysed by Zn-Salen/C.

**Items**			**Total price (million USD)**
Depreciation			-5.0
Utility cost			-4.2
Maintenance cost			-5.0
Labor cost			-5.0
Chemicals	Weight per ton$_{PET}$ (ton)	Unit price (USD ton^{-1})	
Waste PET	1.000	333.9	-33.4
Sodium hydroxide (NaOH)	0.416	340.0	-14.1
Sulfuric acid (H_2SO_4)	0.510	92.7	-4.7
Terephthalic acid (TPA)	0.864	803.7	69.4
Ethylene glycol (EG)	0.323	865.5	28.0
Sodium sulfate (Na_2SO_4)	0.738	69.0	5.0
**Net profit**			**31.0**

Table S3. Simplified techno-economic analysis based on a capability of 70000 tons of PET waste per year for traditional alkaline hydrolysis in 1.0 M NaOH.

**Items**			**Total price (million USD)**
Depreciation			-7.3
Utility cost			-6.1
Maintenance cost			-7.3
Labor cost			-5.0
Chemicals	Weight per ton_{PET} (ton)	Unit price (USD ton^{-1})	
Waste PET	1.000	333.9	-23.4
Sodium hydroxide (NaOH)	0.456	340.0	-10.8
Sulfuric acid (H_2SO_4)	0.560	92.7	-3.6
Terephthalic acid (TPA)	0.864	803.7	48.6
Ethylene glycol (EG)	0.323	865.5	19.6
Sodium sulfate (Na_2SO_4)	0.738	69.0	3.5
**Net profit**			**8.2**

5. References

1. W. M. Ren, Z. W. Liu, Y. Q. Wen, R. Zhang and X. B. Lu, *J. Am. Chem. Soc.*, 2009, **131**, 11509–11518.
2. W. M. Ren, X. Zhang, Y. Liu, J. F. Li, H. Wang and X. B. Lu, *Macromolecules*, 2010, **43**, 1396–1402.
3. T. Uekert, M. F. Kuehnel, D. W. Wakerley and E. Reisner, *Energy Environ. Sci.*, 2018, **11**, 2853–2857.
4. T. Uekert, H. Kasap and E. Reisner, *J. Am. Chem. Soc.*, 2019, **141**, 15201–15210.
5. G. Kresse and J. Furthmiiller, *Comp. Mater. Sci.*, 1996, **6**, 15–50.
6. G. Kresse and J. Furthmüller, *Phys. Rev. B*, 1996, **54**, 11169–11186.
7. P. E. Blöchl, *Phys. Rev. B*, 1994, **50**, 17953–17979.
8. G. Kresse and D. Joubert, *Phys. Rev. B*, 1999, **59**, 1758–1775.
9. J. P. Perdew, K. Burke and M. Ernzerhof, *Phys. Rev. Lett.*, 1996, **77**, 3865–3868.
10. S. Grimme, J. Antony, S. Ehrlich and H. Krieg, *J. Chem. Phys.*, 2010, **132**, 154104.
